# A broad genomic panel of microsatellite loci from *Brycon orbignyanus* (Characiformes: Bryconidae) an endangered migratory Neotropical fish

**DOI:** 10.1038/s41598-018-26623-x

**Published:** 2018-05-31

**Authors:** Gabriel M. Yazbeck, Rafael Sachetto Oliveira, José Mauro Ribeiro, Raíssa D. Graciano, Rosiane P. Santos, Fausto M. S. Carmo, Dominique Lavenier

**Affiliations:** 1 Universidade Federal de São João Del Rei, Departamento de Zootecnia, Laboratório de Recursos Genéticos, Praça Frei Orlando, 170, CEP 36.307-352 São João del-Rei, MG Brazil; 2 Universidade Federal de São João Del Rei, Departamento de Ciência da Computação, Praça Frei Orlando, 170, CEP 36.307-352 São João del-Rei, MG Brazil; 3 Université de Rennes, INRIA, CNRS, IRISA, Rennes, France

**Keywords:** Genome assembly algorithms, Conservation genomics

## Abstract

A broad panel of tens of thousands of microsatellite loci is unveiled for an endangered piracema (*i.e*. migratory) South American fish, *Brycon orbignyanus*. Once one of the main fisheries resources in the Platine Basin, it is now almost extinct in nature and focus of intense aquaculture activity. A total of 178.2 million paired-end reads (90 bases long) were obtained through the use of sequencing-by-synthesis (from a primary genomic library of 500 bp DNA fragments) and is made available through NCBI’s Sequence Read Archive, SRA accession SRX3350440. Short reads were assembled *de novo* and screening for perfect microsatellite motifs revealed more than 81 thousands unique microsatellite loci, for which primer pairs were proposed. A total of 29 polymorphic microsatellite markers were already previously validated for this panel. A partial genomic assembly is hereby presented and these genomic resources are publicly made available. These data will foster the rapid development of hundreds of new DNA markers for genetic diversity studies, conservation initiatives and management practices for this important and depleted species. The availability of such preliminary genomic data will also be of use in the areas of bioinformatics, ecology, genetics and evolution.

## Introduction

Piracema is the seasonal upstream reproductive migratory run undergone by millions of medium and large-sized Neotropical freshwater fish, from different species in South America^1^. These potamodromous species are heavily impacted by pollution, loosely regulated fisheries, species introduction, suppression of riverine vegetation and an intense hydroelectric exploitation regime in many parts of the Platine Basin, the second largest in the continent^2^, encompassing Argentina, Bolivia, Brazil, Paraguay and Uruguay. The successive impoundment of its major rivers lead to deleterious effects across the ichthyofauna: habitat loss (*e.g*. seasonal floodplain lagoons used as natural rearing environments for juveniles), altered landscapes, flood regimes and fish communities. Most conspicuously for migratory species, there is a potential for heavy fragmentation, impeding the long migrations characteristic from the piracema runs^3^.

*Brycon orbignyanus* (Valenciennes, 1850) is a threatened piracema fish from the Platine Basin. It is a medium-sized species with insectivorous-omnivorous dietary habits affected by pollution, deforestation of riparian vegetation, overfishing and damming^4^. Once one of the most prominent fisheries resources in the Platine Basin, it is now almost extinct in the wild, being listed as endangered in the Red book of Brazilian fauna threatened with extinction^5^. Extant populations have been presumably maintained mostly by means of broodstocking practices by environmental hatchery operations, which constitute one of the main mitigation efforts to counteract stocks decline, despite the traditional absence of proper planning and clear definition of goals^6^. These practices still lack adequate efficiency evaluations in the area, as demonstrated elsewhere^7^ and have historically ignored genetic guidelines to reduce inbreeding, random genetic drift, selection to captivity and other potential relevant genetic effects^8^. The shortfall of a deeper understanding of this species’ genetic structure (despite some first efforts^9^) hampers the goal-oriented planning of strategies for effective broodstoking initiatives in *B. orbignyanus*. Thus, the rapid development of molecular markers as tools for the investigation and management of these fish stocks is urgent.

The innovations in DNA sequencing methods during the first decade of the 21st Century have catapulted the *de novo* development of molecular markers for non-model species to new peaks^10^. The first microsatellite loci described for this species were recently unveiled^11^, with empirically validated data from the genomic resources first full and publicly presented herein. Therefore, we hereby unveil a broad panel of potentially amplifiable characterized microsatellite loci in the endangered piracema fish *Brycon orbignyanus*.

## Results

A total of 16.04 Gb (*i.e*. gigabases or 1 × 10^9^ DNA bases) of filtered data was obtained, represented in 178,212,428 paired-end reads, 90 bases long, grouped in two parallel FASTQ files (Supplementary Data S1 stored at NCBI’s Sequence Read Archive - SRA - https://www.ncbi.nlm.nih.gov/sra/SRX3350440). The quality scoring system was the Illumina 1.5, which uses Phred +64 scheme, with characters ranging from “@” to “j”. A total of 97.73% of the data showed a quality value of Q ≥ 20, and the average quality per read was around Q = 38. The GC content of these reads was 41.11%.

A potential amplifiable microsatellite panel was produced for *B. orbignyanus* and made available through the Figshare online data repository (Supplementary Table S2 - 10.6084/m9.figshare.5661988). It consists of a genome-wide characterization table, with 81,241 unique perfect simple sequence repeat loci (di- through hexanucleotides) and shows, among other information, the locus ID (Borb#), microsatellite motif, candidate forward and reverse primers, expected PCR (Polymerase Chain Reaction) products and position over two alternative *de novo* assemblies. Given the lack of the original assembly A0 (Assembly 0), made by service provider BGI (see Methods), we resorted to build alternative assemblies with the filtered short reads and arbitrarily settled with A1 (Assembly 1), created with k-mer = 55 (Supplementary Data S3 - accessible through Figshare - 10.6084/m9.figshare.5661802). The known PCR products expected from the missing assembly A0 were mapped back to A1. The average estimated PCR product length was 146.2 (±21.5) bp. Motif abundance is described in detail in Table 1. Dinucleotides were almost three times more abundant than all the other motif classes together. This panel permitted the rapid empiric validation of the first 29 polymorphic microsatellite markers for *B. orbignyanus*, out of 50 assayed candidates (Borb01-Borb50) and these results alone were previously published^11^.

**Table 1:** Absolute and relative frequency (percentage) of different perfect microsatellite motifs (-nucleotides) found in *B. orbignyanus*.

Motif class	Count	%
Di-	60,295	74.22
Tri-	9,430	11.61
Tetra-	8,820	10.86
Penta-	1,514	1.86
Hexa-	1,182	1.45
Total	81,241	100

The final size of the genomic assembly A1 is 1,113,754,917 bp (including unknown base calls and gaps, N) and 1,039,212,289 bp (not counting Ns – Δ = 74,542,628). A total of 1,273,306 contigs or scaffolds were obtained, the shortest being 100 bp and the longest 172,138 bp (average = 874). Only 55 scaffolds were longer than 100 kbp; 1.97% longer than 10 kbp; 11% longer than 1 kbps and 18.09% of 500 bp or more. The A1 assembly had the CG content of 41.18% and *N*_50_ = 8,463.

We examined all expected PCR product sequences from the target microsatellite loci determined from the A0 assembly, using BLAST (and then using SWIPE, if it failed to retrieve a match) against the resulting A1 assembly. Still missing A0 microsatellite loci were then searched in A1, using forward and reverse primers as queries, with SWIPE. This procedure allowed us to retrieve and locate 97.51% of loci discovered in A0 back to A1. These results are detailed in Table 2, with exact, partial or missing hits. A total of 2,025 loci from A0 could not be accounted for in A1 according to our criteria, including three previous empirically validated loci (Borb13, Borb34 and Borb35)^11^.

**Table 2:** *B. orbignyanus* microsatellite loci described in A0 and mapped back onto assembly A1, according to BLAST and SWIPE results.

Match	Count	%
100%	63,349	77.98
99-90%	14,230	17.52
89-77%	822	1.01
Primers only	815	1
Missing	2,025	2.49
Total	81,241	100

“Primers only” describes matches found with the simultaneous full presence of both primers pair in the same contig/scaffold.

Mapping the short reads onto the A1 assembly resulted in a paired-end BAM file with 108,435,142 alignments, all being mapped end-to-end and properly paired (*i.e*. within the same scaffold, showing the expected orientation and being separated 500 bp or less). This alignment has a mean coverage per contig of 11.37 (±5.77). A second BAM file containing exclusively singleton alignments, average depth of coverage 5.8 (±18), was also produced. Both files were combined into a single run at SRA (Supplementary Data S4 - https://www.ncbi.nlm.nih.gov/sra/SRX3427716).

## Discussion

The results presented here provide the first genomic resources for *B. orbignyanus* and the Bryconidae family available to the scientific community. The sequencing data were considered of high quality (*i.e*. the average probability of a wrong base call is less than one in 16,000). It will possibly find applications in bioinformatics, where bioinformaticians need to test algorithms and pipelines with real-world data sets, in evolutionary comparative studies and in the description of protein coding genes from this species. It also provides a departing point for the complete genome characterization^12^ in this Neotropical fish. Our main goal achieved here, nevertheless, was to provide a broad resource for rapid microsatellite development, so hundreds of markers can be promptly made available to the conservation and aquaculture initiatives for this threatened migratory species. More thorough molecular diversity surveys will allow, for instance, the urgent assessment whether this species is critically impoverished genetically, for it seems to exhibit sudden population booms in adequate environmental conditions^9^, it has recently diminished in certain parts of the Platine Basin and it has likely experienced several bottlenecks due to broodstocking practices. All these scenarios favour strong action of random genetic drift and thus, given this species’ delicate conservation status, it inspires a strong need for focused and intensive studies and directed actions over its potential low genetic variability.

Since we lacked the original service provider’s version of the genomic assembly (A0), we were satisfied with a close but not exactly similar alternative *de novo* assembly (A1), because possible differences in program version used by BGI and our group, along any eventual undisclosed setting of parameters or pipelines would result in slightly divergent genomic assembly outcomes. We were able to successfully track down more than 95% of PCR products inferred from A0 back to genomic assembly A1, with sequence similarity of 90% or more. Unfortunately, around 2.5% of the loci found in the A0 assembly could not be accounted for in A1, according to our criteria, including three previous empirically validated loci^11^. We feel this justifies the salvaging of the unmapped loci from A0 in our final panel (as opposed to their elimination from it), since some of these missing potentially amplifiable loci can knowingly lead to valid PCR results.

The BAM files made available herein constitute a possible departure point for the description of new genes (including those associated with trinucleotide repeats), structural variants and molecular markers in this threatened species and can be used as reference for each individual microsatellite locus selected by other researchers for future validation and development, from this panel. Despite recent promising next-generation sequencing based approaches for surveying genetic diversity^13–16^, traditional microsatellite and other PCR-based marker analyses will still contribute as useful, quick and simple genetic tools to be easily applied^17^ at hatchery and conservation initiatives for *B. orbignyanus*.

This work produced genome-wide resources able to contribute to the rapid and cost-effective development of hundreds of new microsatellite markers, whilst accumulating the first partial genomic data for *B. orbignyanus*. It will certainly foster research, aquaculture and conservation for this species and will likely find application to other diverse areas of biology, evolution and comparative studies.

## Methods

### Specimen collection and DNA extraction

A single individual was used for whole-genome shotgun sequencing. A mature female specimen was captured in the Volta Grande Environmental Station Hatchery, Grande River, MG, Brazil (−20.026197, −48.220430). The fish was euthanized and frozen at around 0 °C, on ice. It was then transported to the laboratory and maintained under −20 °C refrigeration, wrapped in aluminium foil, until DNA extraction. The specimen was thawed at 4 °C overnight and a longitudinal abdominal incision was made with a sterile scalpel for the collection of muscle tissue and gonadal confirmation of the specimen’s sex. This individual is kept as Voucher 120457 from the DNA Bank at the Laboratório de Recursos Genéticos at Universidade Federal de São João Del Rei, LARGE-UFSJ, accessible through www.ufsj.edu.br/recgenlab. The specimen was collected with SISBIO license number 37222-2, following UFSJ’s ethical committee guidelines for animal research (CEUA-UFSJ: *Comissão de Ética no Uso de Animais*, Process 27/2011). No experiments were performed on live specimens.

Total genomic DNA was extracted from ≈2*g* of muscle tissue using Wizard Genomic DNA Purification Kit (Promega, Fitchburg, USA). A total of 6.06 *μg* of good quality DNA was obtained, verified in 1% agarose gel electrophoresis, as a single clear band around 15 kb, only slightly degraded, with a concentration of 233 *μg/μl*, quantified in a Qubit fluorometer. This material was shipped to service providers for downstream treatment.

### Library preparation and sequencing

Library construction, sequencing and first bioinformatics analyses were conducted under the auspices of BGI, Hong Kong/Síntese Biotecnologia, Belo Horizonte, Brazil. Contracted services involved the construction of a single genomic library, sequencing, delivery of raw data and bioinformatics microsatellite search. The short fragments library was constructed according to BGI’s in-house protocols, with random DNA fragmentation and agarose gel purification for genomic fragments with nominal size of 500 bp, followed by adaptor ligation and linear amplification, for the enrichment of fragments with adaptors at both ends. Sequencing was performed over approximately 50% of a single lane from a flow-cell in a HiSeq 2000 equipment (Illumina, San Diego, USA). Sequencing run was January 01, 2014. The library was sequenced targeting an average of 10× coverage depth, for a genome estimated, *a priori*, to be 1.5 Gb. The sequencing assay layout was paired-end with reads 90 bases long.

### Bioinformatics

Service providers performed a bioinformatics pipeline and produced a first table of microsatellite loci and the raw (filtered) short reads data, but delivered no genomic assembly (as per contract), here called A0 (not available): at BGI, short reads were demultiplexed, trimmed for adaptors and filtered by removing reads with quality rate values of Q ≤ 5 in 50% or more bases, and deleting duplicates. The resulting paired-end data were stored in two parallel FASTQ files and used by BGI for *de novo* assembly based on de Bruijn graph using SOAPdenovo 2^18^, with k-mer = 47. The resulting assembly (A0) was screened for perfect simple sequence repeats (microsatellite loci), with repeat motifs ranging from di- through hexanucleotides, with a minimum of five repetitions, except for hexanucleotides which had the minimum number of repeats parameter set as four, with the aid of the SSRIT program^19^. The microsatellite loci sequences found were targeted for primer design with Primer3^20^. Primers were aligned with A0 (using SOAPaligner - http://soap.genomics.org.cn/soapaligner.html), for the retaining of exclusively unique hits. From these results service providers produced a single comprehensive table with characterized microsatellite loci, proposed primers and expected PCR products.

Given the absence of BGI’s assembly (A0), we subsequently evaluated our own *de novo* genomic assembly from the same paired-end short reads, Assembly 1 (A1). We examined it for the presence of partial or exact matches for the microsatellite loci revealed from A0 (represented as expected PCR products retrieved from BGI’s table). We performed the assembly using SOAPdenovo 2 (version 2.04), with default parameters, using k-mer = 55, with the computer cluster at PPGF-UFSJ. We proceeded using BLAST^21^, querying expected PCR products from A0 onto the available A1 assembly. Missing hits were then further searched against A1, using SWIPE^22^. Finally, still unaccounted loci were searched with SWIPE, using forward and reverse primers as independent queries, withholding results where primer pairs were fully detected in the same contig/scaffold. Post-assembly short read alignment with A1 was conducted using SOAPaligner. The resulting SAM files were converted to BAM and analysed with SAMtools^23^ and Tablet^24^. With the joint results from BGI’s table and A1 we produced a final comprehensive panel of genome-wide potentially amplifiable microsatellite loci for *B. orbignyanus*.

### Data availability

The genomic data revealed here are now publicly available from NCBI’s Sequence Read Archive (SRA - https://www.ncbi.nlm.nih.gov/sra) database. Short reads (Supplementary Data S1) can be fetched under Accession https://www.ncbi.nlm.nih.gov/sra/SRX3350440. Supplementary Data S4 with the BAM alignments (made using the genomic assembly A1 as reference) can be retrieved from https://www.ncbi.nlm.nih.gov/sra/SRX3427716.

The proposed genome-wide microsatellite panel for *B. orbignyanus* (Supplementary Table S2 - 10.6084/m9.figshare.5661988) in the “.xlsx” format for Microsoft Excel and the genomic assembly A1 (Supplementary Data S3 - 10.6084/m9.figshare.5661802) in “.fsa” (fasta) format are both available from the Figshare data repository.

## References

[1] Carolsfeld, J. *Migratory fishes of South America: biology, fisheries and conservation status* (IDRC, 2003).

[2] Barletta M (2010). Fish and aquatic habitat conservation in South America: a continental overview with emphasis on neotropical systems. Journal of Fish Biology.

[3] Reis R (2016). Fish biodiversity and conservation in South America. Journal of Fish Biology.

[4] Oliveira DJ, Ashikaga FY, Foresti F, Senhorini JA (2017). Conservation status of the “piracanjuba” *Brycon orbignyanus* (Valenciennes, 1850)(Characiformes, Bryconidae): Basis for management programs. Biodiversidade Brasileira.

[5] Machado, A. B. M., Drummond, G. M. & Paglia, A. P. *Livro vermelho da fauna brasileira ameaçada de extinção* (MMA; Fundação Biodiversitas, 2008).

[6] Agostinho AA, Pelicice FM, Gomes LC, Júlio HF (2010). Reservoir fish stocking: when one plus one may be less than two. Natureza & Conservação.

[7] Attard C (2016). A novel holistic framework for genetic-based captive-breeding and reintroduction programs. Conservation Biology.

[8] DeSalle R, Amato G (2004). The expansion of conservation genetics. Nature Reviews Genetics.

[9] Ashikaga FY, Orsi ML, Oliveira C, Senhorini JA, Foresti F (2015). The endangered species *Brycon orbignyanus*: genetic analysis and definition of priority areas for conservation. Environmental Biology of Fishes.

[10] Ekblom R, Galindo J (2011). Applications of next generation sequencing in molecular ecology of non-model organisms. Heredity.

[11] Arias MC (2016). Microsatellite records for volume 8, issue 1. Conservation Genetics Resources.

[12] Ekblom R, Wolf JB (2014). A field guide to whole-genome sequencing, assembly and annotation. Evolutionary applications.

[13] Davey JW (2011). Genome-wide genetic marker discovery and genotyping using next-generation sequencing. Nature Reviews Genetics.

[14] Peterson BK, Weber JN, Kay EH, Fisher HS, Hoekstra HE (2012). Double digest RADseq: an inexpensive method for *de novo* SNP discovery and genotyping in model and non-model species. PloS one.

[15] Sonah H (2013). An improved genotyping by sequencing (GBS) approach offering increased versatility and efficiency of SNP discovery and genotyping. PloS one.

[16] Shin G (2017). CRISPR–Cas9-targeted fragmentation and selective sequencing enable massively parallel microsatellite analysis. Nature Communications.

[17] Hodel RG (2016). The report of my death was an exaggeration: A review for researchers using microsatellites in the 21^*st*^ century. Applications in plant sciences.

[18] Luo R (2012). SOAPdenovo2: an empirically improved memory-efficient short-read de novo assembler. Gigascience.

[19] Temnykh S (2001). Computational and experimental analysis of microsatellites in rice (*Oryza sativa* l.): frequency, length variation, transposon associations, and genetic marker potential. Genome research.

[20] Untergasser A (2012). Primer3—new capabilities and interfaces. Nucleic acids research.

[21] Boratyn GM (2013). BLAST: a more efficient report with usability improvements. Nucleic acids research.

[22] Rognes T (2011). Faster Smith-Waterman database searches with inter-sequence SIMD parallelisation. BMC bioinformatics.

[23] Li H (2009). The sequence alignment/map format and SAMtools. Bioinformatics.

[24] Milne I (2012). Using Tablet for visual exploration of second-generation sequencing data. Briefings in bioinformatics.

