# Author Correction: A broad genomic panel of microsatellite loci from *Brycon orbignyanus* (Characiformes: Bryconidae) an endangered migratory Neotropical fish

**DOI:** 10.1038/s41598-020-64336-2

**Published:** 2020-04-30

**Authors:** Gabriel M. Yazbeck, Rafael Sachetto Oliveira, José Mauro Ribeiro, Raíssa D. Graciano, Rosiane P. Santos, Fausto M. S. Carmo, Dominique Lavenier

**Affiliations:** 1 Universidade Federal de São João Del Rei, Departamento de Zootecnia, Laboratório de Recursos Genéticos, Praça Frei Orlando, 170, CEP 36.307-352, São João del-Rei, MG Brazil; 2 Universidade Federal de São João Del Rei, Departamento de Ciência da Computação, Praça Frei Orlando, 170, CEP 36.307-352, São João del-Rei, MG Brazil; 3 Université de Rennes, INRIA, CNRS, IRISA, Rennes, France

Correction to: *Scientific Reports* 10.1038/s41598-018-26623-x, published online 31 May 2018

This Article contains typographical errors.

In the Discussion section,

“The sequencing data were considered of high quality (*i.e*. the average probability of a wrong base call is less than one in 16,000).”

should read:

“The sequencing data were considered of high quality (*i.e*. the average probability of a wrong base call is less than one in 6,250).”

In the Acknowledgements section,

“Vicent Wen, PhD”

should read:

“Vicent Wen, MSc”

